# Monitoring for and Management of Endocrine Dysfunction in Adrenoleukodystrophy

**DOI:** 10.3390/ijns8010018

**Published:** 2022-03-02

**Authors:** Isha Kachwala, Molly O. Regelmann

**Affiliations:** 1 Department of Pediatrics, The Children’s Hospital at Montefiore, Albert Einstein College of Medicine, Bronx, NY 10467, USA; ikachwal@montefiore.org; 2 Division of Pediatric Endocrinology and Diabetes, The Children’s Hospital at Montefiore, Albert Einstein College of Medicine, Bronx, NY 10467, USA

**Keywords:** adrenoleukodystrophy, adrenal insufficiency, newborn screening

## Abstract

Adrenoleukodystrophy (ALD) is a peroxisomal disorder affecting the nervous system, adrenal cortical function, and testicular function. Newborn screening for ALD has the potential to identify patients at high risk for life-threatening adrenal crisis and cerebral ALD. The current understanding of the natural history of endocrine dysfunction is limited. Surveillance guidelines for males with ALD were developed to address the unpredictable nature of evolving adrenal insufficiency. Early recognition and management of adrenal insufficiency can prevent adrenal crisis. While testicular dysfunction in ALD is described, the natural history and complications of low testosterone, as well as the management, are not well described.

## 1. Introduction

Adrenoleukodystrophy (ALD) is the most common peroxisomal disorder and has a heterogeneous clinical presentation, impacting the nervous system, adrenal cortical function, and testicular function [1]. New York State initiated newborn screening for ALD on 30 December 2013. Since that time, ALD has been added to the Recommended Uniform Screening Panel, and newborn screening has expanded to twenty-four states and the District of Columbia, as well as to the Netherlands [2,3]. Newborn screening has the benefit of detecting males prior to the onset of symptoms and allowing for therapeutic interventions to prevent the most severe consequences of ALD. Much of the focus of ALD research and reports has been on the neurologic complications. Cerebral ALD presents in about a third of boys during childhood with a rapid loss of cognitive and motor development, leading to complete, devastating disability within two years of initial symptoms. Hematopoietic stem cell transplant and potentially gene therapy can halt the progression of cerebral ALD [4,5]. For those males surviving to adulthood, starting in the second decade of life, adrenomyeloneuropathy (AMN) leads to progressive lower extremity spasticity and bowel and bladder incontinence. The endocrine complications of ALD can also lead to significant morbidity, and in the case of adrenal insufficiency, mortality [1]. The focus of this review is to summarize the screening, presentation, and treatment of the endocrine complications of ALD.

## 2. Pathophysiology of Adrenal Insufficiency

ALD is caused by pathogenic variants to the *ABCD1* gene located at Xq28. The gene encodes the adrenoleukodystrophy protein, an ATP-binding cassette protein necessary for the transport of very long-chain fatty acids (VLCFA) across the peroxisome membrane [1]. The inability to degrade the VLCFA leads to plasma elevations and inclusion in the adrenal cortical and testicular Leydig cells, as well as the nervous system. There are over 940 *ABCD1* non-recurrent variants described in the ALD database (http://adrenoleukodystrophy.info/mutations-and-variants-in-abcd1; last accessed 30 December 2021). There is no known genotype-phenotype correlation, and the degree of elevation in the VLCFA does not correlate with the onset of adrenal insufficiency or neurologic disease [6,7,8]. VLCFA begin depositing in the adrenal cortex during fetal life [9]. They preferentially accumulate in the postnatal zona fasciculata and zona reticularis, which are responsible for the production of glucocorticoids (cortisol) and androgens, respectively. The VLCFA tend to spare the zona glomerulosa, which produces mineralocorticoids. Thus, adrenal insufficiency in ALD tends to present with glucocorticoid and androgen deficiencies, although mineralocorticoid deficiency is described. VLCFA are thought to be directly cytotoxic to adrenocortical cells [10]. An in vitro study also demonstrated VLCFA incorporate into the lipid cell membrane, disrupting adrenocorticotropic hormone (ACTH) binding to its receptor and leading to cortical atrophy [11]. Another proposed mechanism for the glucocorticoid and androgen deficiencies is a relative shortage of cholesterol necessary for their production, as cholesterol is a breakdown product of VLCFA-containing cholesterol esters [12]. The biochemical result of glucocorticoid deficiency is an elevated plasma ACTH and inappropriately low serum cortisol.

## 3. Epidemiology

Historically, ALD was described as an X-linked recessive condition. The most severe presentations of ALD occur in males, but 80% of females with ALD manifest milder myelopathy symptoms by the age of 60 years [13]. Prior to the introduction of newborn screening, the incidence of ALD in the United States was described to be 1:21,000 males and 1:16,800 females [14]. A retrospective study of the US Children’s Hospital Association’s Pediatric Health Information System database noted higher frequencies of *ABCD1* pathogenic variants in those of Latino and African descent, despite higher diagnostic rates in those of non-Hispanic Caucasian descent, suggesting a potential racial bias in diagnosis prior to newborn screening [15]. While *de novo* variants do occur (estimates ~5%), the sex discrepancy, as well as the racial disparities in diagnosis, have been hypothesized to be at least in part due to unrecognized adrenal insufficiency [14,16,17,18]. The published prevalence of ALD based on newborn screening has varied but is consistent with improved identification of those with ALD. In the largest published cohort, California State reported the prevalence of ALD was 1 in 14,397 males and 1 in 9593 females with ~1.85 million newborns screened in the first four years [19]. Minnesota, a relatively small state with potential founder effects, reported an even higher prevalence of 1:4845 in females and 1:3878 in males in the first 67,835 screened newborns [20].

Adrenal insufficiency is reported to occur in the majority of males with ALD. While VLCFA begin to accumulate during the fetal period, there are no reports of adrenal insufficiency at birth. The earliest reports of biochemical adrenal insufficiency are at 5 weeks and 3.5 months of age [21]. California newborn screening reported 14 patients had abnormal ACTH testing in the first three plus years of follow-up and five patients were treated with glucocorticoids [19]. These very early presentations seem to be less common, but more long-term newborn screening follow-up data are needed. The incidence of adrenal insufficiency in males with ALD is estimated to be 80–86%, with a peak onset reported between 3 and 10 years of age [8,22]. In a prospective study of 49 “asymptomatic” boys (average age 4.5 ± 3.5 years) with ALD, 80% had evidence of adrenal dysfunction at baseline and 86% by the end of an average 2 years of follow-up. None of the 18 patients tested had mineralocorticoid deficiency [22]. In a retrospective review of 159 males in ALD neurology clinics, 80% had adrenal insufficiency by the age of 56 years, with a median age of onset of 14 years. Approximately two-thirds of patients had endocrine symptoms at the time they were diagnosed with ALD neurologic disease, and the average diagnostic delay of adrenal insufficiency, based on reported onset of symptoms, was 3.5 years. All of the patients with adrenal insufficiency were treated with glucocorticoid replacement. Mineralocorticoid replacement was prescribed for just over half of the patients, but the median time to treat was 56 years. The study was limited by the retrospective nature and lack of complete laboratory data prior to treatment (only 19 patients had data prior to starting mineralocorticoid therapy, and 4 were started on therapy without evidence of mineralocorticoid deficiency) [8]. There are rare reports of females with ALD developing adrenal insufficiency, and it is estimated that less than 1% develop adrenal insufficiency [23,24].

## 4. Clinical Presentation of Adrenal Insufficiency

Adrenal insufficiency is a challenging clinical diagnosis [25]. Without a known ALD family history prior to newborn screening, adrenal insufficiency symptoms could easily be attributed to other conditions. The presentation of primary adrenal insufficiency can be nonspecific, with symptoms including but not limited to fatigue, anorexia, nausea, stomach upset, and growth failure (typically more so in weight, but if untreated for a prolonged period, may also progress to include growth failure in height). Hyperpigmentation occurs due to increased production of melanocyte-stimulating hormones, a byproduct of ACTH production, and often subtle, particularly in those with darker skin. Classically, hyperpigmentation is noted in the creases of the palms and soles, genitals, areolae, axillae, gums, and the posterior helix of the ears. Axillary and pubic hair loss may be noted after adrenarche [25]. Generalized sparse scalp hair is described in males with ALD and may not be related to androgen deficiency, as the *ABCD1* gene is also expressed in hair follicles [26]. Although less common in ALD, mineralocorticoid deficiency can develop, which will manifest with salt-craving behaviors and, when untreated, is biochemically characterized by hyponatremia, hyperkalemia, mild metabolic acidosis, and elevated plasma renin activity. When untreated, in the setting of acute physical stress (examples include fever, illness, and general anesthesia), primary adrenal insufficiency can lead to an adrenal crisis, a life-threatening state characterized by hypotension, hypoglycemia, and altered mental status [25]. In the absence of mineralocorticoid deficiency, hyponatremia may develop in those with cortisol insufficiency due to a lack of cortisol inhibition of vasopressin secretion [27]. Without recognition of the need for emergent glucocorticoid therapy, cardiovascular collapse can ensure.

## 5. Newborn Screening and Adrenal Insufficiency Surveillance

Newborn screening has allowed for the identification of those at high risk for adrenal insufficiency prior to the onset of symptoms, making it an ideal condition to add to newborn screening panels. Protocols vary among states, but all measure VLCFA from a filter paper specimen and have a confirmatory measurement as a second-tier test. In some states, including New York, *ABCD1* gene sequencing is the third-tier test after confirmation of elevation in the VLCFA [28]. It is assumed that some females with ALD will not be detected, as 15–20% do not have elevations in VLCFA distinguishable from healthy controls [29]. In the Netherlands, an additional step of first identifying male infants is performed, prior to testing for ALD, so as not to identify females, who are only affected in adulthood [3]. Once an infant is identified by newborn screening, most states’ protocols include referral to a metabolic center for confirmatory testing and genetic counseling. ALD is unique to newborn screening in that it is an X-linked condition. It is common for multiple family members to be identified as being at risk for ALD once an infant is diagnosed. Male family members require urgent evaluations for adrenal insufficiency, as well as the cerebral ALD [19,20,28].

VLCFA are also elevated in other rarer peroxisomal disorders, including Zellweger syndrome, acyl CoA oxidase deficiency, and D-bifunctional protein deficiency, and newborn screening can also potentially identify these conditions [28]. Unfortunately, these disorders do not have good therapeutic options for the neurologic conditions, but patients with these conditions are at risk for adrenal insufficiency, which can be treated [30,31,32].

**Figure 1:**
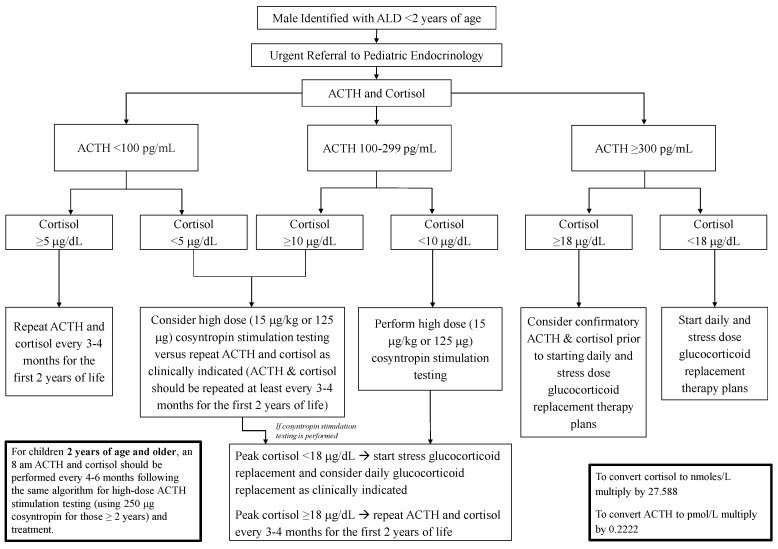
Algorithm for Adrenal Insufficiency Surveillance in Males with ALD. The algorithm attempts to address the lack of clear ACTH and cortisol references ranges. Prior to one year, due to the lack of predictable ACTH and cortisol secretory patterns, it may be reasonable to consider performing a cosyntropin stimulation test as the initial screen if returning for testing is challenging. ALD, adrenoleukodystrophy; ACTH, adrenocorticotropic hormone. Algorithm reprinted with permission [18].

Once confirmed to have ALD, referral to pediatric endocrinology for adrenal insufficiency surveillance should not be delayed given the reports of early biochemical adrenal insufficiency and inability to predict which male infants will develop adrenal insufficiency early [21]. An initial protocol suggested drawing ACTH and cortisol levels every 6 months [28]. Interpreting ACTH and cortisol values during infancy poses challenges, as pulsatile secretion is not predictable in a diurnal pattern due to a lack of mature circadian rhythm. The circadian rhythm likely begins to develop between 1 to 6 months of life but may not be fully established until 3 years of life. Further complicating interpretation of adrenal testing at this age, cortisol levels are generally lower during infancy compared to later in childhood and adulthood. Serum total cortisol measurements are also lower due, in part, to lower corticosteroid-binding globulin production in infants [18,33]. In order to address the unpredictable secretory patterns and weak reference range data for ACTH and cortisol during the first few years of life, The Pediatric Endocrine Society Drug and Therapeutics/Rare Diseases Committee developed a surveillance algorithm for male infants with ALD (Figure 1) [18].

Screening starts at referral, and the recommendation to continue screening every 3–4 months in the first two years was made based on the challenges of diagnosing adrenal insufficiency and the lack of natural history data in the youngest age group. The frequency of screening was spaced to every 4–6 months thereafter. The cutoff values for ACTH and cortisol were based on the Pediatric Endocrine Society 2007 publication [25]. Since that time, there have been significant changes to many cortisol assays, and it has been proposed that cosyntropin stimulated cortisol cutoffs of 14 to 15 μg/dL for monoclonal cortisol antibody immunoassays and LC-MS/MS would reduce false-positive test results [34]. Thus, when making the diagnosis of primary adrenal insufficiency, practitioners must be aware of the assays used and account for age-specific variations.

There are no recommendations for screening females with ALD for adrenal insufficiency, as the reported incidence is low. Females with ALD should be aware of signs and symptoms of adrenal insufficiency and only require testing if there is a clinical suspicion [18].

## 6. Management and Treatment of Adrenal Insufficiency

Treatment of adrenal insufficiency is the same for those with ALD as with other causes of primary adrenal insufficiency [35]. Hydrocortisone is the preferred glucocorticoid, as it is metabolized to cortisol and has the least effect on the epiphyses during childhood. A recent report also noted lower mortality in those with primary adrenal insufficiency treated with hydrocortisone rather than prednisolone [36]. Typical starting hydrocortisone dose is 8–12 mg/m^2^/day divided into three doses, with the higher dose administered in the morning [25,35,37]. Patients with glucocorticoid deficiency should be monitored every 6 months for mineralocorticoid deficiency, and supplementation with fludrocortisones and salt should be administered as clinically indicated [18].

Patients and caregivers should be educated to administer stress doses of hydrocortisone and provided with written instructions in the event of illness or other acute physical stress. Oral hydrocortisone should be increased to 2–3 times physiologic doses administered every 6–8 h, and hydrocortisone sodium succinate 50–100 mg/m^2^/dose intramuscular injection should be administered if unable to take hydrocortisone by mouth in an emergency. Miller, B.S., et al. published a sample “Adrenal Insufficiency Action Plan” and emergency letter, which patients and caregivers should have on hand. Patients should also be instructed to wear a medical identification stating they have adrenal insufficiency and are steroid-dependent [37]. Protocols for stress dosing with sedated procedures and general anesthesia have proven to prevent adrenal crisis [38,39].

## 7. Effects of Investigational Therapies for ALD on Adrenal Function

Screening brain magnetic resonance imaging protocols are used to detect early stages of cerebral ALD in asymptomatic males [40]. Allogeneic hematopoietic stem cell transplant can be an effective treatment for cerebral ALD when performed in those with minimal MRI changes, defined as Loes scores 0.5 to 9 [4]. Hematopoietic stem cell transplant does not alter the progression of adrenal insufficiency [41]. An initial report of autologous CD34+ cells transfected with the elivaldogene tavalentivec (Lenti-D) lentiviral vector also demonstrated the potential to halt cerebral ALD, but the most recent study was held after a patient was reported to have myelodysplastic syndrome. Gene therapy is also not thought likely to stop the progression of adrenal insufficiency [5,18,42]. Both stem cell transplants and gene therapy have the risk of graft failure and infection.

Lorenzo’s oil, a mixture of glyceryl trioleate and glyceryl trierucate, in conjunction with a reduced-fat diet, was ineffective at stopping the progression of cerebral ALD or AMN, but an open-label trial suggested it may delay the development of cerebral ALD in some, but not all, patients [43,44,45,46,47]. A report of seven men with AMN also suggested Lorenzo’s oil may slow the progression of adrenal insufficiency but was only performed over a six-month period [48]. Side effects of Lorenzo’s oil included, but were not limited to, liver dysfunction, thrombocytopenia, gingivitis, and gastrointestinal symptoms [46].

## 8. Testicular Dysfunction

While testicular dysfunction is well documented to occur in men with ALD, there are few reports assessing for the effects of low testosterone. The two largest reports from the 1990s are limited by the quality of the hormone assays and a small number of patients, but in those tested, more than 80% had evidence of primary testicular dysfunction [49,50]. A more recent report of men with AMN from 2012 noted similar fertility compared to the general population despite biochemical evidence of testicular dysfunction in roughly half of the 17 men tested [51]. The mechanisms of testicular dysfunction are similar to those proposed for adrenal insufficiency. VLCFA are cytotoxic to Leydig cells and disrupt the androgen-receptor binding capabilities, leading to atrophy [52]. Testicular dysfunction is confirmed by elevations in gonadotropins (luteinizing hormone and follicular stimulating hormone) and low testosterone levels. Clinically, testicular dysfunction presents as low energy levels/reduced endurance, decreased libido, erectile dysfunction, loss of body hair, depressed mood, decreased muscle mass/strength, and low bone mineral density. Many of these symptoms may also occur due to primary adrenal insufficiency and/or AMN. Teasing out the contribution of testicular dysfunction to symptoms can be challenging [16,53]. There are no reports of the effects of testosterone therapy on men with ALD. A 2003 report of a 3-month cross-over study of 15 patients treated with dehydroepiandrosterone (DHEA) 50 mg daily did not reveal a significant benefit and was associated with lower essential fatty acids; no follow-up studies have been reported [54].

## 9. Conclusions

ALD has the potential to cause significant endocrine morbidity and mortality. Early identification of males with ALD through newborn screening identifies those at high risk for adrenal insufficiency and allows for the initiation of hydrocortisone treatment and prevention of adrenal crisis and chronic symptoms. Adrenal insufficiency surveillance guidance was based on limited cohort studies, and reassessment of the algorithm based on long-term natural history studies will be necessary. Prospective data collection is also necessary to understand the natural history of testicular dysfunction and the potential risks/benefits of therapeutic interventions.

## Abbreviations

ACTH	Adrenocorticotropic hormone
ALD	Adrenoleukodystrophy
AMN	Adrenomyeloneuropathy
DHEA	Dehydroepiandrosterone
VLCFA	Very long-chain fatty acids
